# Investigation of Hydrogen Sulfide Exposure and Lung Function, Asthma and Chronic Obstructive Pulmonary Disease in a Geothermal Area of New Zealand

**DOI:** 10.1371/journal.pone.0122062

**Published:** 2015-03-30

**Authors:** Michael N. Bates, Julian Crane, John R. Balmes, Nick Garrett

**Affiliations:** 1 School of Public Health, University of California, Berkeley, California, United States of America; 2 School of Medicine and Health Sciences, University of Otago, Wellington, New Zealand; 3 Faculty of Health, Auckland University of Technology, Auckland, New Zealand; Institute for Health & the Environment, UNITED STATES

## Abstract

**Background:**

Results have been conflicting whether long-term ambient hydrogen sulfide (H_2_S) affects lung function or is a risk factor for asthma or chronic obstructive pulmonary disease (COPD). Rotorua city, New Zealand, has the world’s largest population exposed to ambient H_2_S—from geothermal sources.

**Objectives:**

We investigated associations of H_2_S with lung function, COPD and asthma in this population.

**Methods:**

1,204 of 1,639 study participants, aged 18–65 years during 2008–2010, provided satisfactory spirometry results. Residences, workplaces and schools over the last 30 years were geocoded. Exposures were estimated from data collected by summer and winter H_2_S monitoring networks across Rotorua. Four metrics for H_2_S exposure, representing both current and long-term (last 30 years) exposure, and also time-weighted average and peak exposures, were calculated. Departures from expected values for pre-bronchodilator lung function, calculated from prediction equations, were outcomes for linear regression models using quartiles of the H_2_S exposure metrics. Separate models examined participants with and without evidence of asthma or COPD, and never- and ever-smokers. Logistic regression was used to investigate associations of COPD (a post-bronchodilator FEV_1_/FVC < 70% of expected) and asthma (doctor-diagnosed or by FEV_1_ response to bronchodilator) with H_2_S exposure quartiles.

**Results:**

None of the exposure metrics produced evidence of lung function decrement. The logistic regression analysis showed no evidence that long-term H_2_S exposure at Rotorua levels was associated with either increased COPD or asthma risk. Some results suggested that recent ambient H_2_S exposures were beneficially associated with lung function parameters.

**Conclusions:**

The study found no evidence of reductions in lung function, or increased risk of COPD or asthma, from recent or long-term H_2_S exposure at the relatively high ambient concentrations found in Rotorua. Suggestions of improved lung function associated with recent ambient H_2_S exposures require confirmation in other studies.

## Introduction

Hydrogen sulfide (H_2_S) is a highly acutely toxic gas with a characteristic “rotten egg” smell at low ppb concentrations. The main toxic effects are believed to be on the nervous system, with respiratory paralysis occurring at concentrations above 500 ppm [1]. If the person affected is not rapidly removed from exposure then death may soon occur. Although the acute toxic effects of H_2_S are reasonably well characterized and accepted, the effects of long-term, lower level exposures (below about 2 ppm) are controversial and not well characterized. Among the toxic effects for which there is still uncertainty about long-term H_2_S exposure is lung function, measured by spirometry. Epidemiologic study results have been mixed. Studies have been carried out in H_2_S-exposed populations living near natural gas refineries [2, 3], paper mills [4], or concentrated animal feeding operations [5, 6] and in sewer workers [7] and workers at an aircraft factory [8]. Some have reported decrements in lung function or residual volume; others have found no evidence of an association. Most of these studies have involved small numbers of participants and interpretation of their results has usually been complicated by the presence of other co-emitted exposures.

Probably the largest population with relatively high long-term ambient exposure to H_2_S are residents of the city of Rotorua (population ∼60,000), which sits on a geothermal field in the Taupo Geothermal Zone of New Zealand’s North Island. Geysers and boiling water and boiling mud pools are situated in and around the city, and the characteristic odor of H_2_S is often apparent. The Rotorua area has been inhabited for centuries by the Maori people and, since the 19^th^ Century, by European immigrants, who used it as a spa. Rotorua has long been considered a particularly useful place to investigate long-term effects of H_2_S [9]. The other geothermal emissions are mostly water vapor and CO_2_, unlikely to confound any H_2_S effects. Most non-geothermal sources of H_2_S tend to co-emit other potentially toxic compounds.

Ecological studies have suggested that there may be health effects related to H_2_S exposure in Rotorua [10–13]. We conducted a cross-sectional study to further investigate such effects. Our previously published results suggest that self-reported, doctor-diagnosed asthma and asthma symptoms are not associated with current exposures to H_2_S [14]; nor did we find evidence of effects on cognitive function [15]. Other objectives of the same study were to investigate whether longer-term H_2_S exposure was associated with reductions in lung function, and/or was a risk factor for chronic obstructive pulmonary disease (COPD), as suggested by some previous studies. These objectives were achieved and in this paper we present the results.

## Methods

### Ethics Statement

Institutional Review Board approvals for the study procedures were obtained from the University of California, Berkeley, for the University of California sites, and from the Northern Ethics Committee in New Zealand. Written informed consent was obtained from all participants before participation.

### Participants

Enrolled were 1637 adults, aged 18–65, who had lived in Rotorua for the last 3 years or longer. Recruitment has been previously described [14]. Briefly, participants were recruited from a comprehensive primary care medical register, using a stratification method that was intended to ensure a balanced distribution of residential H_2_S exposures. Excluded were persons unable to speak and write English, blind people, pregnant women, and those who, because of disability, were unable to visit the study clinic. First, the city was stratified into ‘‘high”, ‘‘medium” and ‘‘low” H_2_S-exposed areas, as indicated by a previous exposure investigation [16], and then potential study participants were obtained equally from these 3 areas by random selection from the central patient register. This initial stratification was to ensure a good variation in participant exposures and was not used further in the main analysis.

Participants attended the study clinic, where they were administered a questionnaire and a series of clinical tests, including spirometry. Height and weight were accurately measured. The questionnaire sought demographics and personal data, as well as residential, school and workplace histories (locations and dates) going back 30 years. The examination site was in a low H_2_S exposure area of Rotorua. Total participation time was on average about 2.5 hours.

### Spirometric Testing

Spirometry was carried out by trained staff using the EasyOne^TM^ spirometer (ndd Medical Technologies, Andover, MA) before and 15 minutes after administration of two puffs of the inhaled bronchodilator, albuterol, following American Thoracic Society/European Respiratory Society performance guidelines [17]. All spirometry curves were reviewed by a trained technician for acceptability and a subset were further reviewed by two experienced physicians (JB and JC).

### Exposure Estimation

We estimated H_2_S concentrations at each participant’s residential, workplace and school locations across Rotorua using data from H_2_S monitoring networks deployed across the city for two-week periods [14]. Data from three monitoring networks—summer and winter, 2010, and winter, 2011, were used to calculate weighted average H_2_S concentrations at each location. To calculate mean concentrations, each of the two winter concentrations was allocated 25% weight and the summer concentration 50% weight, to avoid overweighting the winter results. Two types of H_2_S exposure metric were used: 1) the mean time-weighted average exposure, based on hours at work or school, and assuming the remainder was spent at home; and 2) the maximum average exposure, derived by selecting the higher of the average home, work or school exposure. Exposure metrics were created for both time of participation (‘current exposure’) and for the last 30 years (‘long-term exposure’). Rather than being based on a biological rationale, for which there is yet no good basis, we chose the last 30 years because pre-testing of the questionnaire showed it to be a practical length of time to inquire about.

For assessment of the association with pulmonary function we considered *a priori* that the long-term H_2_S exposure metrics would be most appropriate. These metrics were based on reported residential, workplace and school locations over the 30 years prior to participation, including dates of beginning and ending residence, employment and school attendance, collected by questionnaire. Since actual H_2_S exposure measurements were made only at around the time of the study, the long-term metrics are based on the assumption that the distribution of sources and their emissions remained approximately constant over the previous 3 decades. The two long-term exposure metrics were average yearly H_2_S exposure over the last 30 years and average annual peak (school, workplace or home) exposure across those years. All locations outside Rotorua were assigned a zero H_2_S concentration.

Year-by-year H_2_S exposure estimates were first created for each of the last 30 years, or less for participants younger than 30 years. Using geocoded H_2_S concentrations, plus reported daily hours at work and hours at school, a time-weighted average H_2_S exposure concentration was estimated for each year. Sites (home, work or school) associated with each participant’s highest H_2_S exposure concentration in each year were also identified. For our main analysis we used the averages of the 30-year concentration averages or peaks, including zeroes for years of not living in Rotorua.

### Data Analysis

The EasyOne spirometer contains an algorithm to judge the adequacy and reproducibility of expiratory maneuvers. We used the standard criteria rating scale to judge the adequacy of measurements of FVC (forced vital capacity), FEV_1_ (forced expiratory volume in one second) and FEF_25–75_ (forced expiratory flow 25–75%). Included in our data analysis were grades A (3 acceptable FEV_1_ or FVC measurements within 100 ml), B (3 acceptable FEV_1_ or FVC measurements within 150 ml) and C (2 acceptable FEV_1_ or FVC measurements within 200 ml). All “acceptable” measures were confirmed by visual inspection of the shape of the flow-volume curves. Some attempts judged acceptable by the spirometer were excluded after curve inspection.

Expected values were calculated with the equations recommended by the Global Lung Function Initiative, ERS Task Force [18] and using the associated Global Lungs predicted values calculator (Version 1.3.4 build 3). As input, the prediction equations require age, gender, ethnic group, and height.

Asthma was defined as either self-reported doctor’s diagnosis of asthma or > 12% increase, which was more than 200 ml, in post-bronchodilator FEV_1_ compared with pre-bronchodilator FEV_1_. COPD was defined as a post-bronchodilator FEV_1_/FVC less than 70% of the expected value.

The main analysis employed linear regression of the differences between the observed and the expected values for FEV_1_, FVC, FEV_1_/FVC and FEF_25–75%._ For clarity of presentation, we used a common set of covariates: tobacco smoking (ex-, current, and never-smoker), education (5 levels: no secondary school qualification, secondary school qualification, trade certificate, bachelor’s degree, and post-graduate degree), and income (5 levels). Since the prediction equations do not have a separate ethnicity category for Polynesian (Maori or Pacific Island) subjects, we also included a dichotomous covariate for self-identified Polynesian ethnicity. Separate, parallel analyses were carried out for never- and ever-smokers, participants with and without evidence of asthma, and participants with and without COPD, as judged by post-bronchodilator FEV_1_/FVC. Analysis of possible effect modification between quartiles of H_2_S exposure and smoking, asthma and COPD was carried out using interaction terms in multiple linear regression analyses.

Because of the possibility that long-term exposure measures might contain misclassifications that obscured any associations, we also carried out a parallel analysis with the current exposure measures that have been previously described [14].

To further investigate the relationship with COPD, we carried out an unconditional logistic regression analysis confined to participants with satisfactory post-bronchodilator spirometry results, based on the long-term exposure measures. Covariates used for adjustment were sex, age, ethnicity, education, income and smoking status. A parallel analysis using the current exposure measures was also performed.

## Results

Based on the last recorded telephone number from medical records, attempts were made to contact 6,573 people. Of these, 4,498 people were contacted, 976 of whom were ineligible. Of the remainder, 1,927 (54.7%) agreed to participate, but because of study field work timeframe limitations, only 1,639 people actually participated, during 2008–10.

Study participants had lived in Rotorua between 3 and 64 years (median 18 years). The median H_2_S concentration for current residences was 20.3 ppb, with mean 20.8 ppb (standard deviation (SD), 15.5 ppb). For current workplaces, the median and mean (SD) were 26.4 and 27.0 (17.7) ppb, respectively. The range for both residences and workplaces was 0–64 ppb.

Table 1 presents covariate data for study participants, by quartile of the time-weighted, long-term exposure metric, restricted to the 1,204 participants with satisfactory pre-bronchodilator spirometry performance. Corresponding data for current exposures have previously been published [14].

**Table 1:** Description of the study population, by quartiles (Q1 to Q4) of estimated average time-weighted H_2_S exposure concentration over the last 30 years for study participants who performed pre-bronchodilator spirometry to a satisfactory standard, Rotorua, New Zealand, 2008–2010.

	N	Q1	Q2	Q3	Q4
**H**_**2**_ **S concentration range**		(0–6 ppb)	(7–11 ppb)	(12–18 ppb)	(19–58 ppb)
**N**	1,204	304	313	293	294
Sex:					
Female	790 (65.6%)	66.8%	67.4%	62.8%	65.3%
Male	414 (34.4%)	33.2%	32.6%	37.2%	34.7%
Age Group (years):					
18–29	105 (8.7%)	10.2%	11.8%	5.1%	7.5%
30–39	236 (19.6%)	27.3%	22.0%	17.1%	11.6%
40–49	331 (27.5%)	31.3%	26.2%	31.7%	20.8%
50–59	354 (29.4%)	21.4%	25.9%	32.4%	38.4%
60+	178 (14.8%)	9.9%	14.1%	13.7%	21.8%
Ethnic Group:					
European	960 (79.7%)	79.3%	76.0%	84.0%	79.9%
Maori	203 (16.9%)	16.1%	19.2%	14.0%	18.0%
Other	41 (3.4%)	4.6%	4.8%	2.1%	2.0%
Education:					
No qualification earned	143 (11.9%)	14.8%	12.1%	9.2%	11.2%
Secondary school qualification	28 (22.3%)	19.4%	19.8%	23.9%	26.2%
Non-degree or trade qualification	460 (38.2%)	39.5%	36.7%	38.6%	38.1%
Bachelor’s degree	218 (18.1%)	17.8%	19.8%	18.4%	16.3%
Graduate degree	115 (9.6%)	8.6%	11.5%	9.9%	8.2%
Income (NZ$):					
$0-$20K	261 (21.7%)	33.8%	21.4%	16.3%	15.3%
>$20K-$40K	359 (29.8%)	28.1%	29.6%	31.6%	29.9%
>$40K-$60K	416 (34.6%)	25.8%	38.2%	35.8%	38.5%
>$60K-$80K	62 (5.2%)	5.7%	5.3%	4.2%	5.6%
>$80K	74 (6.2%)	4.4%	4.0%	9.3%	6.9%
Don't Know/Refused	32 (2.7%)	2.3%	1.6%	2.9%	3.8%
Tobacco Smoking Status:					
Never	614 (51.0%)	52.6%	53.0%	51.9%	46.3%
Ex	359 (29.8%)	26.6%	24.0%	33.5%	35.7%
Current	231 (19.2%)	20.7%	23.0%	14.7%	18.0%
Asthma ever:					
No	851 (70.7%)	65.8%	70.9%	72.0%	74.2%
Yes	353 (29.3%)	34.2%	29.1%	28.0%	25.9%
COPD (spirometric evidence)					
No	1,093 (90.8%)	90.8%	89.8%	93.9%	88.8%
Yes	111 (9.2%)	9.2%	10.2%	6.1%	11.2%

Abbreviations:N, number of participants; NZ$, New Zealand dollars; ppb, parts per billion, Q, quartile.

Some small differences across long-term H_2_S exposure quartiles are evident, such as a slightly older population in the highest exposure quartile, but there are no major differences between quartiles. A similar pattern for asthma as that previously found using current H_2_S exposure metrics [14] is evident for asthma (either from self-reported doctor’s diagnosis or spirometric evidence from this study)—a monotonic trend of lower prevalence with increasing H_2_S exposure. Similar patterns are evident for the exposure metric involving the average of peak exposures. However, this metric has a more even distribution of asthma prevalence across the exposure quartiles. No trends are evident for COPD. Table 2 shows for the long-term exposure metrics results of the linear regression analysis of the differences between the observed spirometric parameters (FEV_1_, FEV_1_/FVC, FEF_25–75%_) and those expected from the prediction equations. Separate analyses were run for both exposure metrics and separately for those with evidence of ever having had asthma and those with no evidence for asthma, for those with and without post-bronchodilator evidence of COPD, and for ever-smokers and never-smokers. The column for quartile 1 (Q1) shows the mean difference from the expected value for that quartile and the values for quartiles 2 to 4 (Q2 to Q4) show the adjusted mean differences relative to Q1. Overall, there is no clear evidence of any association with long-term H_2_S exposure. There are some monotonic trends, but they represent small differences and may simply be random variation.

**Table 2:** Linear regressions of differences between observed and expected spirometric parameters (pre-bronchodilator spirometry results), showing adjusted differences from the first quartile (Q) of long-term (30 year) H_2_S exposure concentration in ppb, after controlling for covariates.

Test	N^a^	Time-weighted mean exposure.				Maximum exposure at work, home, or school.			
		Q1	Q2	Q3	Q4	Q1	Q2	Q3	Q4
		(0–6 ppb)	(7–11 ppb)	(12–18 ppb)	(19–58 ppb)	(0–10 ppb)	(11–20 ppb)	(21–31 ppb)	(32–60 ppb)
		Mean	Difference from Q1			Mean	Difference from Q1		
		(SE)^b^	(95% confidence interval) ^c^			(SE)^b^	(95% confidence interval) ^c^		
*FEV*_*1*_ *(liters)*									
All participants	1,204	−0.29	0.01	0.07	0.04	−0.28	0.02	0.06	0.00
		(0.04)	(−0.09, 0.10)	(−0.03, 0.17)	(−0.06, 0.14)	(0.04)	(−0.07, 0.12)	(−0.04, 0.15)	(−0.10, 0.10)
No asthma^d^	714	−0.20	−0.03	0.04	0.03	−0.19	0.00	0.02	−0.01
		(0.05)	(−0.14, 0.08)	(−0.07, 0.16)	(−0.08, 0.14)	(0.05)	(−0.11, 0.12)	(−0.09, 0.13)	(−0.13, 0.10)
Asthma^d^	353	−0.48	0.03	0.03	0.03	−0.50	0.01	0.13	0.02
		(0.08)	(−0.16, 0.22)	(−0.17, 0.23)	(−0.17, 0.24)	(0.09)	(−0.19, 0.21)	(−0.07, 0.33)	(−0.19, 0.22)
Never-smokers	614	−0.20	−0.01	0.06	0.00	−0.18	−0.02	0.03	−0.05
		(0.05)	(−0.14, 0.12)	(−0.07, 0.19)	(−0.14, 0.13)	(0.05)	(−0.15, 0.11)	(−0.10, 0.16)	(−0.19, 0.08)
Ever-smokers	590	−0.40	0.04	0.10	0.09	−0.39	0.08	0.08	0.07
		(0.06)	(−0.10, 0.18)	(−0.04, 0.25)	(−0.05, 0.23)	(0.06)	(−0.07, 0.22)	(−0.06, 0.22)	(−0.07, 0.321)
No COPD^e^	917	−0.18	−0.05	0.02	0.03	−0.17	−0.04	0.01	0.00
		(0.04)	(−0.15, 0.04)	(−0.08, 0.12)	(−0.07, 0.13)	(0.04)	(−0.14, 0.05)	(−0.08, 0.11)	(−0.09, 0.10)
COPD^e^	111	−1.17	0.28	0.05	0.26	−1.13	0.30	0.14	0.10
		(0.13)	(−0.09, 0.64)	(−0.38, 0.48)	(−0.11, 0.62)	(0.13)	(−0.10, 0.70)	(−0.24, 0.52)	(−0.28, 0.47)
*FVC* *(liters)*									
All participants	1,204	−0.17	−0.01	0.06	0.02	−0.18	0.04	0.07	0.00
		(0.04)	(−0.11, 0.10)	(−0.05, 0.17)	(−0.09, 0.13)	(0.04)	(−0.07, 0.15)	(−0.04, 0.18)	(−0.11, 0.11)
No asthma^d^	714	−0.11	−0.03	0.06	0.04	−0.12	0.04	0.05	0.01
		(0.06)	(−0.16, 0.11)	(−0.08, 0.19)	(−0.10, 0.17)	(0.05)	(−0.09, 0.18)	(−0.08, 0.19)	(−0.13, 0.14)
Asthma^d^	353	−0.28	−0.03	0.01	0.01	−0.30	−0.04	0.12	−0.03
		(0.08)	(−0.24, 0.18)	(−0.21, 0.22)	(−0.21, 0.23)	(0.09)	(−0.26, 0.17)	(−0.10, 0.33)	(−0.25, 0.20)
Never-smokers	614	−0.10	−0.03	0.03	−0.01	−0.10	0.00	0.03	−0.05
		(0.06)	(−0.18, 0.12)	(−0.12, 0.19)	(−0.17, 0.14)	(0.06)	(−0.15, 0.15)	(−0.12, 0.18)	(−0.21, 0.11)
Ever-smokers	590	−0.25	0.03	0.10	0.05	−0.25	0.09	0.10	0.02
		(0.07)	(−0.13, 0.19)	(−0.06, 0.27)	(−0.11, 0.21)	(0.07)	(−0.07, 0.25)	(−0.06, 0.26)	(−0.11, 0.21)
No COPD^e^	917	−0.10	−0.07	0.02	0.01	−0.09	−0.04	0.03	−0.02
		(0.05)	(−0.18, 0.05)	(−0.10, 0.13)	(−0.11, 0.13)	(0.05)	(−0.16, 0.07)	(−0.09, 0.14)	(−0.14, 0.10)
COPD^e^	111	−0.73	0.18	0.05	0.20	−0.82	0.47	0.25	0.12
		(0.16)	(−0.26, 0.63)	(−0.47, 0.57)	(−0.24, 0.64)	(0.16)	(−0.01, 0.95)	(−0.21, 0.70)	(−0.32, 0.56)
*FEV*_*1*_ /*FVC*									
All participants	1,204	−0.05	0.00	0.01	0.01	−0.05	0.00	0.00	0.00
		(0.00)	(−0.01, 0.01)	(−0.01, 0.02)	(−0.01, 0.02)	(0.00)	(−0.01, 0.02)	(−0.01, 0.01)	(−0.01, 0.01)
No asthma^d^	714	−0.04	0.00	0.00	0.00	−0.03	0.00	0.00	0.00
		(0.01)	(−0.01, 0.01)	(−0.01, 0.01)	(−0.01, 0.01)	(0.00)	(−0.01, 0.01)	(−0.02, 0.01)	(−0.02, 0.01)
Asthma^d^	353	−0.09	0.01	0.02	0.00	−0.09	0.02	0.01	0.01
		(0.01)	(−0.01, 0.04)	(−0.01, 0.04)	(−0.02, 0.03)	(0.01)	(−0.01, 0.05)	(−0.02, 0.03)	(−0.02, 0.03)
Never-smokers	614	−0.04	0.01	**0.01**	0.00	−0.03	0.00	0.00	0.00
		(0.01)	(−0.01, 0.02)	**(0.00, 0.03)**	(−0.01, 0.01)	(0.01)	(−0.01, 0.01)	(−0.01, 0.02)	(−0.02, 0.01)
Ever-smokers	590	−0.07	0.00	0.01	**0.01**	−0.07	0.01	0.00	0.01
		(0.01)	(−0.01, 0.02)	(−0.01, 0.03)	**(0.00, 0.03)**	(0.01)	(−0.01, 0.03)	(−0.02, 0.02)	(−0.01, 0.03)
No COPD^e^	917	−0.04	0.00	0.00	**0.01**	−0.03	0.00	0.00	0.00
		(0.00)	(−0.01, 0.01)	(−0.01, 0.01)	**(0.00, 0.02)**	(0.00)	(−0.01, 0.01)	(−0.01, 0.01)	(−0.01, 0.01)
COPD^e^	111	−0.21	0.04	0.03	0.03	−0.20	0.03	0.00	0.03
		(0.02)	(−0.01, 0.09)	(−0.03, 0.09)	(−0.02, 0.08)	(0.02)	(−0.02, 0.09)	(−0.05, 0.05)	(−0.02, 0.08)
*FEF*_*25–75%*_ *(liters/sec)*									
All participants	1,204	−0.51	−0.01	0.10	0.11	−0.48	−0.03	0.05	0.05
		(0.06)	(−0.15, 0.14)	(−0.04, 0.25)	(−0.04, 0.25)	(0.06)	(−0.18, 0.11)	(−0.09, 0.20)	(−0.10, 0.19)
No asthma^d^	712	−0.31	−0.09	0.05	0.05	−0.29	−0.06	0.01	−0.02
		(0.07)	(−0.23, 0.09)	(−0.13, 0.22)	(−0.12, 0.23)	(0.07)	(−0.23, 0.11)	(−0.18, 0.17)	(−0.20, 0.16)
Asthma^d^	353	−0.90	0.09	0.07	0.10	−0.93	0.00	0.13	0.19
		(0.10)	(−0.18, 0.36)	(−0.21, 0.34)	(−0.19, 0.38)	(0.11)	(−0.28, 0.28)	(−0.15, 0.41)	(−0.09, 0.48)
Never-smokers	614	−0.35	−0.02	0.12	0.00	−0.28	−0.14	0.01	−0.07
		(0.08)	(−0.22, 0.18)	(−0.08, 0.33)	(−0.21, 0.21)	(0.08)	(−0.35, 0.06)	(−0.19, 0.22)	(−0.28, 0.14)
Ever-smokers	590	−0.69	0.01	0.13	**0.22**	−0.68	0.08	0.07	0.19
		(0.08)	(−0.20, 0.22)	(−0.09, 0.34)	**(0.01, 0.42)**	(0.08)	(−0.14, 0.29)	(−0.14, 0.28)	(−0.02, 0.40)
No COPD^e^	915	−0.37	−0.06	0.05	0.13	−0.33	−0.10	0.02	0.08
		(0.06)	(−0.21, 0.09)	(−0.10, 0.21)	(−0.03, 0.29)	(0.06)	(−0.25, 0.06)	(−0.14, 0.17)	(−0.08, 0.24)
COPD^e^	111	−1.78	0.25	0.03	0.20	−1.69	0.10	0.06	0.03
		(0.09)	(−0.05, 0.55)	(−0.32, 0.38)	(−0.10, 0.50)	(0.10)	(−0.23, 0.44)	(−0.26, 0.38)	(−0.28, 0.34)

Results where the null value is excluded from the confidence interval are in bold.

^a^ N, number of participants in the model

^b^ Unadjusted values for the differences between the observed value and the expected value from the prediction equation.

^c^ Adjusted for education, income, Polynesian race, and tobacco smoking.

^d^ Further restricted to participants with either acceptable post-bronchodilator results or a doctor’s diagnosis of asthma.

^e^. Further restricted to participants with acceptable post-bronchodilator results

The parallel analysis using the current exposure metrics showed evidence of differences between the lowest exposure quartile and the higher exposure quartiles. Virtually all results showed evidence of better lung function in the higher exposure quartiles compared to the lowest exposure quartile (Table 3). Where the null value was excluded from the confidence interval (i.e., p < 0.05), results are shown in bold font.

**Table 3:** Linear regressions of differences between observed and expected spirometric parameters (pre-bronchodilator spirometry results), showing adjusted differences from the first quartile (Q) of current H_2_S exposure concentration in ppb, after controlling for covariates.

Test	N^a^	Time-weighted mean exposure.				Maximum exposure at work, home, or school.			
		Q1	Q2	Q3	Q4	Q1	Q2	Q3	Q4
		(0–10 ppb)	(11–20 ppb)	(21–30 ppb)	(31–64 ppb)	(0–17 ppb)	(18–29 ppb)	(30–44 ppb)	(45–64 ppb)
		Mean	Difference from Q1			Mean	Difference from Q1		
		(SE)^b^	(95% confidence interval) ^c^			(SE)^b^	(95% confidence interval) ^c^		
*FEV*_*1*_ *(liters)*									
All participants	1,204	−0.35	**0.15**	0.08	**0.11**	−0.32	0.07	0.04	0.07
		(0.04)	**(0.05, 0.24)**	(−0.02, 0.18)	**(0.01, 0.21)**	(0.04)	(−0.02, 0.17)	(−0.06, 0.13)	(−0.03, 0.17)
No asthma^d^	714	−0.32	**0.20**	**0.18**	**0.13**	−0.27	**0.13**	0.09	0.05
		(0.05)	**(0.09, 0.31)**	**(0.07, 0.29)**	**(0.01, 0.24)**	(0.05)	**(0.01, 0.24)**	(−0.02, 0.20)	(−0.06, 0.17)
Asthma^d^	353	−0.46	0.02	−0.06	0.06	−0.48	0.02	−0.01	0.08
		(0.07)	(−0.19, 0.22)	(−0.25, 0.13)	(−0.14, 0.26)	(0.07)	(−0.18, 0.22)	(−0.21, 0.19)	(−0.12, 0.27)
Never-smokers	614	−0.29	**0.15**	0.09	0.10	−0.21	0.08	0.01	−0.06
		(0.04)	**(0.02, 0.28)**	(−0.04, 0.22)	(−0.04, 0.23)	(0.05)	(−0.05, 0.21)	(−0.12, 0.14)	(−0.19, 0.08)
Ever-smokers	590	−0.42	0.13	0.08	0.11	−0.42	0.06	0.07	**0.19**
		(0.06)	(−0.02, 0.27)	(−0.06, 0.23)	(−0.03, 0.25)	(0.06)	(−0.09, 0.21)	(−0.07, 0.21)	**(0.05, 0.33)**
No COPD^e^	917	−0.26	**0.13**	**0.10**	0.07	−0.23	**0.12**	0.03	0.05
		(0.04)	**(0.04, 0.23)**	**(0.00, 0.20)**	(−0.02, 0.17)	(0.04)	**(0.02, 0.22)**	(−0.07, 0.12)	(−0.05, 0.15)
COPD^e^	111	−1.11	0.16	0.01	0.29	−1.18	0.25	0.19	0.35
		(0.11)	(−0.22, 0.54)	(−0.37, 0.39)	(−0.05, 0.64)	(0.12)	(−0.13, 0.62)	(−0.25, 0.64)	(−0.01, 0.71)
*FVC* *(liters)*									
All participants	1,204	−0.23	**0.15**	0.06	0.10	−0.21	0.10	0.03	0.07
		(0.04)	**(0.04, 0.26)**	(−0.05, 0.17)	(−0.01, 0.21)	(0.05)	(−0.01, 0.21)	(−0.08, 0.14)	(−0.04, 0.18)
No asthma^d^	714	−0.22	**0.20**	**0.16**	0.11	−0.19	**0.17**	0.08	0.07
		(0.06)	**(0.07, 0.34)**	**(0.02, 0.29)**	(−0.02, 0.25)	(0.06)	**(0.03, 0.31)**	(−0.05, 0.22)	(−0.07, 0.21)
Asthma^d^	353	−0.28	0.01	−0.08	0.08	−0.32	0.04	0.00	0.10
		(0.07)	(−0.21, 0.23)	(−0.29, 0.13)	(−0.14, 0.29)	(0.08)	(−0.18, 0.25)	(−0.21, 0.22)	(−0.12, 0.31)
Never-smokers	614	−0.20	**0.17**	0.08	0.09	−0.13	0.11	0.00	−0.07
		(0.05)	**(0.17, 0.32)**	(−0.07, 0.24)	(−0.07, 0.24)	(0.06)	(−0.15, 0.15)	(−0.15, 0.15)	(−0.23, 0.09)
Ever-smokers	590	−0.26	0.13	0.03	0.10	−0.28	0.07	0.06	**0.21**
		(0.07)	(−0.04, 0.29)	(−0.13, 0.19)	(−0.06, 0.26)	(0.07)	(−0.09, 0.24)	(−0.10, 0.21)	**(0.05, 0.36)**
No COPD^e^	917	−0.17	**0.13**	0.08	0.06	−0.15	**0.12**	0.03	0.05
		(0.05)	**(0.01, 0.25)**	(−0.04, 0.20)	(−0.06, 0.18)	(0.05)	**(0.00, 0.24)**	(−0.09, 0.14)	(−0.07, 0.17)
COPD^e^	111	−0.70	0.20	−0.13	0.30	−0.79	0.27	0.13	0.40
		(0.12)	(−0.25, 0.66)	(−0.58, 0.33)	(−0.11, 0.71)	(0.14)	(−0.18, 0.73)	(−0.40, 0.67)	(−0.03, 0.83)
*FEV*_*1*_ /*FVC*									
All participants	1,204	−0.056	0.009	0.009	0.008	−0.053	0.001	0.007	0.002
		(0.005)	(−0.002, 0.021)	(−0.002, 0.021)	(−0.003, 0.020)	(0.005)	(−0.011, 0.012)	(−0.004, 0.018)	(−0.009, 0.014)
No asthma^d^	714	−0.045	0.011	0.013	0.009	−0.039	−0.002	0.006	−0.001
		(0.006)	(−0.002, 0.024)	(−0.000, 0.026)	(−0.004, 0.022)	(0.006)	(−0.015, 0.011)	(−0.007, 0.019)	(−0.014, 0.012)
Asthma^d^	353	−0.079	−0.001	0.000	−0.004	−0.08	−0.001	−0.000	−0.004
		(0.009)	(−0.027, 0.024)	(−0.025, 0.024)	(−0.030, 0.022)	(0.009)	(−0.027, 0.024)	(−0.026, 0.025)	(−0.029, 0.021)
Never-smokers	614	−0.042	0.008	0.007	0.008	−0.036	−0.000	0.005	0.000
		(0.005)	(−0.006, 0.022)	(−0.007, 0.021)	(−0.006, 0.023)	(0.005)	(−0.014, 0.014)	(−0.009, 0.019)	(−0.015, 0.015)
Ever-smokers	590	−0.070	0.009	0.015	0.006	−0.068	0.002	0.010	0.005
		(0.007)	(−0.010, 0.028)	(−0.003, 0.034)	(0.012, 0.025)	(0.007)	(−0.017, 0.022)	(−0.008, 0.028)	(−0.014, 0.023)
No COPD^e^	917	−0.039	0.007	0.009	0.007	−0.036	0.004	0.001	0.002
		(0.004)	(−0.002, 0.016)	(−0.000, 0.018)	(−0.003, 0.016)	(0.004)	(−0.005, 0.014)	(−0.008, 0.010)	(−0.008, 0.011)
COPD^e^	111	−0.191	−0.011	0.019	0.003	−0.201	0.011	0.021	0.008
		(0.019)	(−0.063, 0.040)	(−0.033, 0.071)	(−0.050, 0.044)	(0.020)	(−0.041, 0.062)	(−0.040, 0.081)	(−0.041, 0.057)
*FEF*_*25–75%*_ *(liters/sec)*									
All participants	1,201	−0.62	**0.21**	**0.17**	**0.19**	−0.53	0.06	0.05	0.08
		(0.05)	**(0.06, 0.35)**	**(0.02, 0.32)**	**(0.05, 0.34)**	(0.06)	(−0.09, 0.21)	(−0.10, 0.19)	(−0.07, 0.23)
No asthma^d^	712	−0.50	**0.24**	**0.29**	0.16	−0.39	0.11	0.10	−0.05
		(0.07)	**(0.07, 0.42)**	**(0.12, 0.47)**	(−0.02, 0.33)	(0.07)	(−0.07, 0.29)	(−0.08, 0.27)	(−0.23, 0.13)
Asthma^d^	352	−0.90	0.09	0.00	0.14	−0.88	−0.00	0.05	0.15
		(0.09)	(−0.19, 0.38)	(−0.26, 0.27)	(−0.15, 0.42)	(0.10)	(−0.28, 0.27)	(−0.33, 0.23)	(−0.13, 0.43)
Never-smokers	613	−0.48	0.19	0.17	0.20	−0.35	0.05	0.03	−0.04
		(0.07)	(−0.01, 0.40)	(−0.03, 0.38)	(−0.01, 0.41)	(0.08)	(−0.15, 0.25)	(−0.18, 0.23)	(−0.26, 0.18)
Ever-smokers	588	−0.75	0.20	0.20	0.17	−0.71	0.08	0.08	0.19
		(0.08)	(−0.01, 0.42)	(−0.01, 0.41)	(0.03, 0.38)	(0.08)	(−0.14, 0.30)	(−0.13, 0.29)	(−0.02, 0.40)
No COPD^e^	915	−0.50	**0.22**	**0.22**	0.16	−0.42	**0.19**	0.06	0.07
		(0.06)	**(0.07, 0.38)**	**(0.07, 0.38)**	(−0.00, 0.31)	(0.06)	**(0.03, 0.035)**	(−0.09, 0.21)	(−0.09, 0.23)
COPD^e^	110	−1.77	0.09	0.06	**0.33**	−1.79	0.16	0.22	**0.31**
		(0.08)	(−0.21, 0.40)	(−0.25, 0.37)	**(0.05, 0.61)**	(0.09)	(−0.15, 0.47)	(−0.14, 0.58)	**(0.01, 0.60)**

Results where the null value is excluded from the confidence interval are in bold.

^a^ N, number of participants in the model.

^b^ Unadjusted values for the differences between the observed value and the expected value from the prediction equation.

^c^ Adjusted for education, income, Polynesian race, and tobacco smoking.

^d^ Further restricted to participants with either acceptable post-bronchodilator results or a doctor’s diagnosis of asthma.

^e^. Further restricted to participants with acceptable post-bronchodilator results.

Using interaction terms in multiple linear regression models, we further investigated the possibility of effect modification between H_2_S exposure and smoking, asthma, and COPD statuses, each dichotomized, and after splitting our study population into those below and those above the median age (48.1 years). Table 4 shows results for the older half of our population, using the long-term, time-weighted average exposure metric. Measures for spirometric parameters are the differences between the dichotomized categories of smoking, asthma and COPD, after adjustment for the range of covariates used. The table provides some suggestion that increasing H_2_S exposure mitigates lung damage in older ever-smokers relative to older never-smokers. No such patterns are evident for asthma or COPD. A generally similar, but less pronounced, pattern was evident with the long-term peak H_2_S exposure metric. No such patterns were apparent when the same analysis was carried out for the younger half of the participants, although the differences in spirometric parameters between ever-smokers and never-smokers were much smaller in this group than in the older participants (results not shown).

**Table 4:** Adjusted differences between ever- and never-smokers, people with and without asthma, and people with and without COPD, by quartile of long-term (30 year) time-weighted average H_2_S exposure.

Spirometric measure	H_2_S Quartile	Difference (liters)^a^		
		Smoking^b^	Asthma^c^	COPD^c^
**FEV**_**1**_	**1**	−0.36	−0.36	−0.97
	**2**	−0.24	−0.38	−0.66
	**3**	−0.20	−0.29	−0.77
	**4**	−0.11	−0.34	−0.72
	*P for trend*	*0*.*09*	*0*.*75*	*0*.*30*
**FVC**	**1**	−0.33	−0.26	−0.64
	**2**	−0.19	−0.31	−0.37
	**3**	−0.08	−0.28	−0.35
	**4**	−0.10	−0.28	−0.34
	*P for trend*	*0*.*15*	*0*.*99*	*0*.*23*
**FEV**_**1**_/**FVC**	**1**	−0.050	−0.078	−0.190
	**2**	−0.033	−0.062	−0.146
	**3**	−0.040	−0.029	−0.155
	**4**	−0.013	−0.051	−0.156
	*P for trend*	*0*.*07*	*0*.*10*	*0*.*19*
**FEF**_**25–75**_	**1**	−0.43	−0.62	−1.41
	**2**	−0.34	−0.62	−1.17
	**3**	−0.52	−0.32	−1.29
	**4**	−0.12	−0.53	−1.34
	*P for trend*	*0*.*18*	*0*.*44*	*0*.*99*

^a^ (measure with condition, e.g., ever-smoking)–(measure without condition e.g., never-smoking).

^b^ Adjusted for age, sex, income, education, and Polynesian race.

^c^ Adjusted for age, sex, income, education, Polynesian race, and smoking status.

We carried out a parallel analysis of possible effect modification using the current exposure metrics. However, it showed no clear evidence of associations with any of smoking, asthma or COPD (results not shown).

Table 5 shows the adjusted logistic regression results for COPD, using both long-term and current exposure metrics. It shows no evidence of an association with H_2_S exposure, but associations with other covariates, especially sex, age, ethnicity and smoking, were clear and in the expected directions. A similar logistic regression analysis using the long-term metrics was carried out for asthma. It showed no clear evidence of associations. Using the current H_2_S exposure metrics provided evidence of associations in a protective direction for asthma, but not for COPD (Table 6).

**Table 5:** Unconditional logistic regression analysis of COPD and both long-term (up to 30-years) and current H_2_S exposure metrics in Rotorua city residents, 2008–2010, limited to participants with satisfactory post-bronchodilator measurements (N = 1,185).

H_2_ S exposure quartile ^a^	Time-weighted mean exposure				Maximum exposure at work, home, or school			
	COPD cases (%)	Non-cases (%)	OR	95% CI	COPD cases (%)	Non-cases (%)	OR	95% CI
**Long-term (30 years) exposure** ^**b**^								
1 (Reference)	32 (10.6)	269 (89.4)	1.00	-	32 (11.0)	260 (89.0)	1.00	-
2	34 (11.4)	264 (88.6)	0.91	0.52, 1.58	28 (9.52)	266 (90.5)	0.85	0.47, 1.53
3	23 (7.96)	266 (92.0)	0.69	0.38, 1.27	29 (9.39)	280 (90.6)	0.86	0.48, 1.55
4	36 (12.1))	261 (87.9)	0.98	0.56, 1.70	36 (12.4)	254 (87.6)	1.03	0.58, 1.82
**Current exposure** ^**c**^								
1 (Reference)	38 (12.9)	256 (87.1)	1.00	-	36 (12.2)	260 (87.8)	1.00	-
2	24 (7.9)	280 (92.1)	0.59	0.33, 1.07	34 (11.9)	245 (88.2)	1.06	0.61, 1.84
3	33 (11.0)	268 (89.0)	0.90	0.53, 1.55	24 (7.4)	299 (92.6)	0.70	0.38, 1.27
4	30 (10.5)	256 (89.5)	0.69	0.40, 1.21	31 (11.1)	248 (88.9)	0.93	0.53, 1.63
TOTAL	125 (10.6)	1,060 (89.5)			125 (10.6)	1,060 (89.5)		

a Adjusted for sex, age-group, ethnicity, education, income, smoking status.

b For H_2_S exposure concentrations, see Table 2.

c For H_2_S exposure concentrations, see Table 3.

**Table 6:** Unconditional logistic regression analysis of asthma and both long-term (up to 30-years) and current H_2_S exposure metrics in Rotorua city residents, 2008–2010, limited to participants with satisfactory pre- and post-bronchodilator measurements (N = 1,098).

H_2_ S exposure quartile ^a^	Time-weighted mean exposure				Maximum exposure at work, home, or school			
	Asthma cases (%)	Non-cases (%)	OR	95% CI	Asthma cases (%)	Non-cases (%)	OR	95% CI
**Long-term (30 years) exposure** ^**b**^								
1 (Reference)	113 (40.2)	168 (59.8)	1.00	-	101 (37.1)	171 (62.9)	1.00	-
2	100 (35.0)	186 (65.0)	0.82	0.58, 1.17	102 (36.7)	176 (63.3)	0.97	0.67, 1.39
3	90 (33.5)	179 (66.5)	0.76	0.51, 1.27	96 (33.2)	193 (66.8)	0.92	0.64, 1.33
4	81 (30.9)	181 (69.1)	0.76	0.52, 1.11	85 (32.8)	174 (67.2)	1.00	0.68, 1.47
**Current exposure** ^**c**^								
1 (Reference)	116 (43.6)	150 (53.4)	1.00	-	114 (43.2)	150 (56.8)	1.00	-
2	85 (29.9)	199 (70.1)	0.58	0.40, 0.84	89 (32.8)	182 (67.2)	0.69	0.48, 1.00
3	97 (34.6)	183 (65.4)	0.75	0.52, 1.07	92 (31.3)	202 (68.7)	0.66	0.46, 0.95
4	86 (32.1)	182 (67.9)	0.66	0.46, 0.95	89 (33.1)	180 (66.9)	0.68	0.47, 0.99
TOTAL	384 (35.0)	714 (65.0)			384 (35.0)	714 (65.0)		

a Adjusted for sex, age-group, ethnicity, education, income, smoking status.

b For H_2_S exposure concentrations, see Table 2.

c For H_2_S exposure concentrations, see Table 3.

## Discussion

This is by far the largest study that has examined pre- and post-bronchodilator lung function or COPD in relation to ambient H_2_S exposure. For all participants combined and all subgroups, our results show no evidence of an adverse association between the ambient H_2_S levels found in Rotorua and any of the spirometric parameters examined or COPD. In that sense, the spirometry results are consistent with some other studies of H_2_S-exposed populations, which found no evidence of an association with H_2_S [2–5]. Other studies have suggested H_2_S is associated with decrements in lung function. Richardson [7] found reduced FEV_1_/FVC levels in non-smoking sewer workers compared with water treatment workers. However, this study is difficult to interpret because of the multiple exposures that sewer workers experience. Kilburn [6] reported reduced FVC and FEV_1_ in 25 people living near concentrated animal (swine) feeding operations in Ohio compared with a total of 58 people in two reference groups, one of which was in Tennessee. This study is open to major suspicion of selection bias, since the “exposed” group was self-organizing. This may have led to aggregation of a group with a high prevalence of ill health, potentially unrelated to living near animal feeding operations. There is also the interpretive problem that emissions from such animal operations contain more than just H_2_S.

An interesting and unexpected result was found when we carried out an analysis of possible effect modification between H_2_S and older study participants separately dichotomized by smoking, asthma and COPD statuses. This produced some suggestion that long-term H_2_S exposure might mitigate lung damage in smokers, although the association was not clearly evident in those with COPD (Table 4). This result is interesting because there is some evidence from animal studies that hydrogen sulfide is associated with amelioration of tobacco smoke-induced damage [19, 20]. However, the result would need replication in other epidemiologic studies before it could be given much weight.

Although our prior expectation had been that the long-term exposure metrics would be the most appropriate and informative, based on an assumption of a cumulative effect, the most consistent evidence of associations with H_2_S was found with the current exposure metrics, which we previously used to investigate doctor-diagnosed asthma [14]. These associations were found with both the linear and the logistic regression analyses (Tables 3 and 6). The current study included both previously diagnosed asthma cases and those whom we found had spirometric evidence of asthma. That we found evidence solely of protective associations with asthma is consistent with our earlier study of doctor-diagnosed asthma and asthma symptoms in the same study population [14]. However, the present study was limited to participants with satisfactory pre-bronchodilator spirometry results. It included 31 who were judged asthmatic solely on the basis of their spirometric results and considered non-asthmatic in the previous analysis.

Of particular interest are our COPD results. Our logistic regression analysis (Table 5) showed no evidence of an adverse association with H_2_S exposure, although odds ratios for the higher exposure quartiles were consistently below 1.0. However, a few other epidemiologic studies, some in Rotorua, have suggested that H_2_S is associated with COPD. In an ecologic study based on hospital discharge data, Bates et al. [12] obtained a standardized incidence ratio (SIR) of 1.57 (95% CI: 1.32, 1.86) for COPD and allied conditions associated with living in high H_2_S exposure areas of Rotorua. This study was limited to considering residential area only and had very limited ability to consider potential confounding exposures. In a later ecologic study using Rotorua hospital data, Durand and Wilson [13] reported relative risk estimates of 5.1 to 6.1 for COPD in clusters “spatially coincident with the geothermal field”. However, as individual identifiers were not available in the hospital admissions data, multiple hospital visits by the same individual could not be excluded.

A cross-sectional study of 4,735 Norwegian famers, obtained an odds ratio of 1.4 (95% CI: 1.1, 1.7) for “high” H_2_S exposure relative to “low” H_2_S exposure [21]. Personal task-specific exposures to H_2_S and a number of chemical and biological agents were obtained from a random sample of 290 farms and approximately 30 samples were collected for each task and agent. Many of the exposures were highly correlated. A particular limitation of that study was that COPD was defined using the lower limit of normal of the pre-bronchodilator FEV_1_/FVC ratio, as a bronchodilator was not administered to study participants. Although individuals with known current asthma were excluded, this could have led to an over-estimation of the prevalence of COPD.

That H_2_S might cause COPD is plausible, since it is a reactive and irritant gas and can be oxidized to sulfuric acid. However, the relationship between H_2_S and COPD is likely to be more complicated than that: H_2_S has been shown to be an endogenously produced “gasotransmitter”, with anti-inflammatory and cytoprotective functions [22]. These properties have led to exploration of its use for protection against ventilator-induced lung injury [23, 24]. Research into the role H_2_S plays in the pathogenesis of lung diseases, including COPD and asthma, is ongoing [25]. Adding plausibility to our findings that recent H_2_S exposures are associated with better lung function, recently published mouse and human tissue studies have shown H_2_S to have airway smooth muscle relaxing properties [26–28].

It is important to address the question of why we found evidence of associations with H_2_S, based on the current exposure metrics, but much less so with the long-term exposure metrics. There are two obvious possible reasons: (1) as discussed below, there may be too much exposure misclassification in the long-term H_2_S metrics; or (2) it may be that the lung function benefits of H_2_S are related mainly to recent exposure. Either or both of these possibilities may have played a role. Although misclassification of the long-term exposures almost certainly played a role, that there was some evidence of mitigation of smoking-induced lung function reduction (Table 4) suggests that the long-term H_2_S exposure estimates may be adequate for detection of some associations.

Potential limitations of our study in terms of selection bias, information bias and confounding need to be considered. These have previously been discussed [14] and are only summarized here. Selection bias could have occurred if the exposure pattern of participants was different to that of non-participants. As we had data on age, sex, ethnic group and exposure area of the non-participants, we were able to investigate this. That women and older people were the most willing to participate, is consistent with findings of many other epidemiologic studies [29]. As other New Zealand studies have found [30, 31], Maori and Pacific Island people were less likely to participate than people of European ethnicity. However, we found no indication that participation in our study was differential by H_2_S exposure status of the current residence. This suggests selection bias related to willingness to participate is not likely to have been a problem.

Spirometric outcomes were measured using standard methods and field staff were thoroughly trained in the use of the spirometer, with extensive oversight by two pulmonary physicians during the data collection phase. Spirometry results used in the data analysis were checked to ensure that they met acceptability criteria. Therefore, misclassification of spirometric parameters is unlikely to be a substantive source of error.

The potential for exposure misclassification is of more concern. A key assumption inherent in our 30-year exposure metrics is that there was little change in H_2_S sources over that period. This assumption is not unreasonable, since the distribution of geothermal features generating H_2_S has changed only slowly in Rotorua. Despite this, there must have been exposure misclassification because we computed our estimates only on the basis of when and where participants resided, worked, and attended school. Also, we relied substantially on their memories to identify these places, and earlier locations may be subject to greater misclassification, which may have been more of a problem with older participants. There will also have been some misclassification associated with interpolating exposures between monitoring sites by the kriging method. As exposure misclassification is not likely to be associated with outcome status, we expect it would have tended to attenuate any actual exposure-response relationships.

Uncontrolled confounding is always a possibility in any epidemiologic study, although we are unaware of any other factors that impact lung function that are likely to be correlated with H_2_S exposure in Rotorua.

In conclusion, we believe this has been the largest and most detailed epidemiologic study ever to have investigated effects on lung function of relatively high, long-term, ambient H_2_S concentrations. It had a population-based participant selection process, objective and quality-controlled measures of outcome, absence of potentially confounding co-pollutants, and a comprehensive modeling of long-term H_2_S exposures, although these exposures were estimated from H_2_S measurements collected in 2010/11. Most importantly, the results provide no evidence that chronic H_2_S exposure, at the ambient levels found in and around Rotorua, is associated with impairment of pulmonary function, and no evidence that it is associated with COPD. The results provide some epidemiologic evidence that recent ambient H_2_S exposures may benefit lung function, possibly through airways smooth muscle relaxation, although these results require confirmation in other epidemiologic studies.
